# *In vitro* toxicity of different-sized ZnO nanoparticles in Caco-2 cells

**DOI:** 10.1186/1556-276X-8-496

**Published:** 2013-11-21

**Authors:** Tianshu Kang, Rongfa Guan, Xiaoqiang Chen, Yijuan Song, Han Jiang, Jin Zhao

**Affiliations:** 1 Zhejiang Provincial Key Laboratory of Biometrology and Inspection and Quarantine, China Jiliang University, Hangzhou 310018, People’s Republic of China; 2 Hubei University of Technology, Wuhan 430068, People’s Republic of China

**Keywords:** ZnO nanoparticles, Nanotoxicity: Caco-2 cells

## Abstract

There has been rapid growth in nanotechnology in both the public and private sectors worldwide, but concern about nanosafety exists. To assess size-dependent cytotoxicity on human cancer cells, we studied the cytotoxic effect of three kinds of zinc oxide nanoparticles (ZnO NPs) on human epithelial colorectal adenocarcinoma (Caco-2) cells. Nanoparticles were first characterized by size, distribution, and intensity. Multiple assays have been adopted to measure the cell activity and oxidative stress. The cytotoxicity of ZnO NPs was time dependent and dose dependent. The 24-h exposure was chosen to confirm the viability and accessibility of the cells and taken as the appropriate time for the following test system. The IC_50_ value was found at a low concentration. The oxidative stress elicited a significant reduction in glutathione with increase in reactive oxygen species and lactate dehydrogenase. The toxicity resulted in a deletion of cells in the G1 phase and an accumulation of cells in the S and G2/M phases. One type of metallic oxide (ZnO) exerted different cytotoxic effects according to different particle sizes. Data from the previous experiments showed that 26-nm ZnO NPs appeared to have the highest toxicity to Caco-2 cells. The study demonstrated the toxicity of ZnO NPs to Caco-2 cells and the impact of particle size, which could be useful in the medical applications.

## Background

ZnO nanoparticles with a unique optical, electrical, and thermal performance have been widely used in the field of catalysis, sunscreen cosmetics, paint materials, and food packaging materials [1,2]. The chemical and physical properties of nanoparticles have a strong influence on the way they interact with biological components or the environment [3] and also on the way they move, accumulate, and clear in the body [4,5]. Industrial food processing is intended to modify flavor, texture, and storage behavior by mixing with zinc oxide nanoparticles (ZnO NPs). After ingestion of food containing ZnO NPs, mechanical (chewing and peristalsis) and chemical (interaction with intestinal enzymes) processes reduce food into smaller components to maintain physiological processes. Much research has shown that ZnO NPs cause cytotoxicity to many types of cells, such as osteoblast cancer cells [6], human bronchial epithelial cells (BEAS-2B) [7], human kidney cells [8], human alveolar adenocarcinoma cells [9], human hepatocytes, and embryonic kidney cells [10]. Relevant studies report that ZnO nanoparticles primarily cause disease to organs including the stomach and intestines. Human epithelial colorectal adenocarcinoma (Caco-2) cell lines are a continuous line of heterogeneous epithelial colorectal adenocarcinoma cells as a confluent monolayer. *In vitro* measurements are not only rapid and easy to perform, but are also used to predict *in vivo* toxicity. In *in vivo* experiments, the dose is an important factor in mice. Changes in low doses could not be detected, and high concentrations of ZnO nanoparticles cause acute death.

ZnO NPs are also considered as one of the most toxic NPs with the lowest LD_50_ value among the engineered metal oxide nanoparticles in many references [11-13]. Wang has demonstrated that the ranking of the toxicity of metal oxides to the test cells is as follows: TiO_2_ < Co_3_O_4_ < ZnO < CuO [14]. Kao et al. surmised the mechanical toxicological pathway of ZnO NPs. The cytosolic entrance and dissolution of ZnO NPs lead to an initial elevation in cytosolic Zn^2+^. Mitochondria sequester excess cytosolic Zn^2+^, resulting to a rise in mitochondrial Zn^2+^. High Zn^2+^ in the mitochondria induces mitochondrial membrane potential collapse, which activates caspase-3 and leads to cell apoptosis and lactate dehydrogenase (LDH) release [15,16]. Reactive oxygen species (ROS) are produced as a normal product of cellular metabolism. In particular, one major contributor to oxidative damage is hydrogen peroxide (H_2_O_2_), which is converted from superoxide that leaks from the mitochondria. However, under oxidative stress conditions, excessive ROS can damage cellular proteins, lipids, and DNA, leading to fatal lesions in the cell. In summary, the 3-(4,5-dimethylthiazol-2-yl)-2,5-diphenyltetrazolium bromide (MTT) assay was used to evaluate cellular toxicity. ROS production, glutathione (GSH) detection, and LDH leakage were assessed in intracellular oxidative conditions. In this study, we report that one type of metallic oxide (ZnO) exerted different cytotoxic effects according to different particle sizes. The results were mainly correlated with particle sizes.

## Methods

### Characterization of particles

ZnO NPs were purchased from Hangzhou Wan Jing New Limited (Hangzhou, China). The mother liquid was diluted with phosphate-buffered saline (PBS) to become 400 μg/ml in ultrasound before exposure (amplitude 100%, pulse 5 s/10 s, 2 min). The suspension of ZnO nanoparticles was prepared (6.25, 12.5, 25, 50, and 100 μg/ml) in a DMEM serum-free medium without l-glutamin and antibiotics. The nanoparticles were tested with anhydrous ethanol ultrasonic dispersion using a support film containing the copper mesh fish sample to dry at room temperature to characterize NPs with transmission electron microscopy (JEOL JEM-2100, JEOL Ltd., Tokyo, Japan). Zetasizer instrumentation (Malvern Instruments, Worcestershire, UK) was used to analyze the intensity and size of the particles.

### Cell cultures

Caco-2 cells (CBCAS, Shanghai, China) were cultured in DMEM medium (Gibco BRL, Gaitherburg, MD, USA), with 10% fetal calf serum (Sijiqing Company, Hangzhou, China), 2.9 μg/ml l-glutamine, 1 μg/ml streptomycin, and 100 units/ml penicillin (Sigma Chemicals, Balcatta, WA, USA). The cells were cultured at 37°C in water-saturated air supplemented with 5% CO_2_ and passaged twice a week. At 80% confluence, the cells were harvested using 0.25% trypsin and were subcultured into 75-cm^2^ flasks, 6-well plates, 24-well plates, or 96-well plates according to the selection of experiments.

### Cell activity

Twenty thousand cells were seeded into every well of a 96-well plate. Cells were treated with the described particle suspensions (0, 6.25, 12.5, 25, 50, and 100 μg/ml) for 12, 24, and 36 h. Cytotoxicity was determined by measuring the enzymatic reduction of yellow tetrazolium MTT to a purple formazan, as measured at 570 nm using an enzyme-labeled instrument. The results are given as relative values to the negative control in percentage, whereas the untreated (positive) control is set to be 100% viable. The percentage of cell proliferation was calculated as [17]

$$\mathrm{MTT}\;\mathrm{cell}\;\mathrm{activity}\%=\frac{A_{\exp}-A_{\mathrm{neg}}}{A_{\mathrm{con}}-A_{\mathrm{neg}}}$$

where *A*_exp_ is the amount of experimental group absorbance, *A*_neg_ is the amount of blank group absorbance, and *A*_con_ is the amount of control group absorbance.

### Oxidative stress damage

#### ROS assay

ROS was monitored by measurement of hydrogen peroxide generation. In brief, cells were seeded (20,000 cells per well) in the 96-well plates. Then, the serum-free medium with ZnO NPs was removed for 24 h, and the medium was renewed with DCF-DA dissolved in the medium for 30 min. After washing twice with the serum-free medium, the intensity of DCF-DA fluorescence was determined by using ELISA (Tecan, Grödig, Austria).

#### GSH detection

Cells were collected by centrifugation at 400 × *g* for 5 min at 4°C. The supernatant was removed. The suspension was washed and centrifuged two times using cold PBS to remove all traces of the medium. The cell pellet was sonicated at 300 W (amplitude 100%, pulse 5 s/10 s, 2 min) to obtain the cell lysate. A cell suspension of 600 μl, reaction buffer solution of 600 μl, and substrate solution of 150 μl were transferred to a fresh tube. The standard group was 25 μM GSH dissolved in GSH buffer solution. The blank group was replaced by PBS. The absorbance was read at 405 nm using a microplate reader. Protein content was measured with the method of Bradford using BSA as the standard.

#### LDH assay

Cells were seeded (1 million cells per well) in 6-well plates. Cells were treated with a range of concentrations of ZnO NPs for 24 h. Plates were centrifuged at 400 × *g* for 5 min, and the supernatant was transferred from each well to the corresponding well of the 96-well test plate. For each well, a total of 60 μl of reaction mixture was prepared: 2 μl sodium, 2 μl INT, 20 μl substrate, and 36 μl PBS; the reaction was incubated at 37°C for 30 min. The absorbance was read at 450 nm with an ELISA plate reader.

### AO/EB double staining

Caco-2 cells were plated in a 12-well plate exposed to the concentrations of 12.5 and 50 μg/ml ZnO NPs for 24 h. After completion of the exposure period, cells were washed with PBS. Adding 300 μl PBS containing 100 μg/ml acridine orange and 100 μg/ml ethidium bromide (Sigma), we examined dyeing results using a fluorescence microscope (Nikon Eclipse Ti, Nikon, Shinjuku, Tokyo, Japan).

### Flow assay

Caco-2 cells were plated in a 6-well plate and exposed at concentrations of 12.5 and 50 μg/ml ZnO NPs for 24 h. Cell suspension was harvested and fixed in 70% ethanol at 4°C during 24 h. The suspension was washed and centrifuged two times using cold PBS to remove all traces of ethanol. Cells were suspended in 100 μl PBS, and 10 μl RNase A solution was added. The tubes were incubated at 37°C for 30 min. An equal volume (110 μl) of propidium iodide (PI) was added to each tube and incubated at 4°C for at least 30 min. The tubes were diluted using 280 μl PBS and measured by flow cytometry (FC500Mel, Beckman Coulter Ltd., Brea, CA, USA).

### Statistical analysis

The data were expressed as mean ± SD of three independent experiments. SPSS 16.0 software was used for the statistical analysis.

## Results

**Figure 1:**
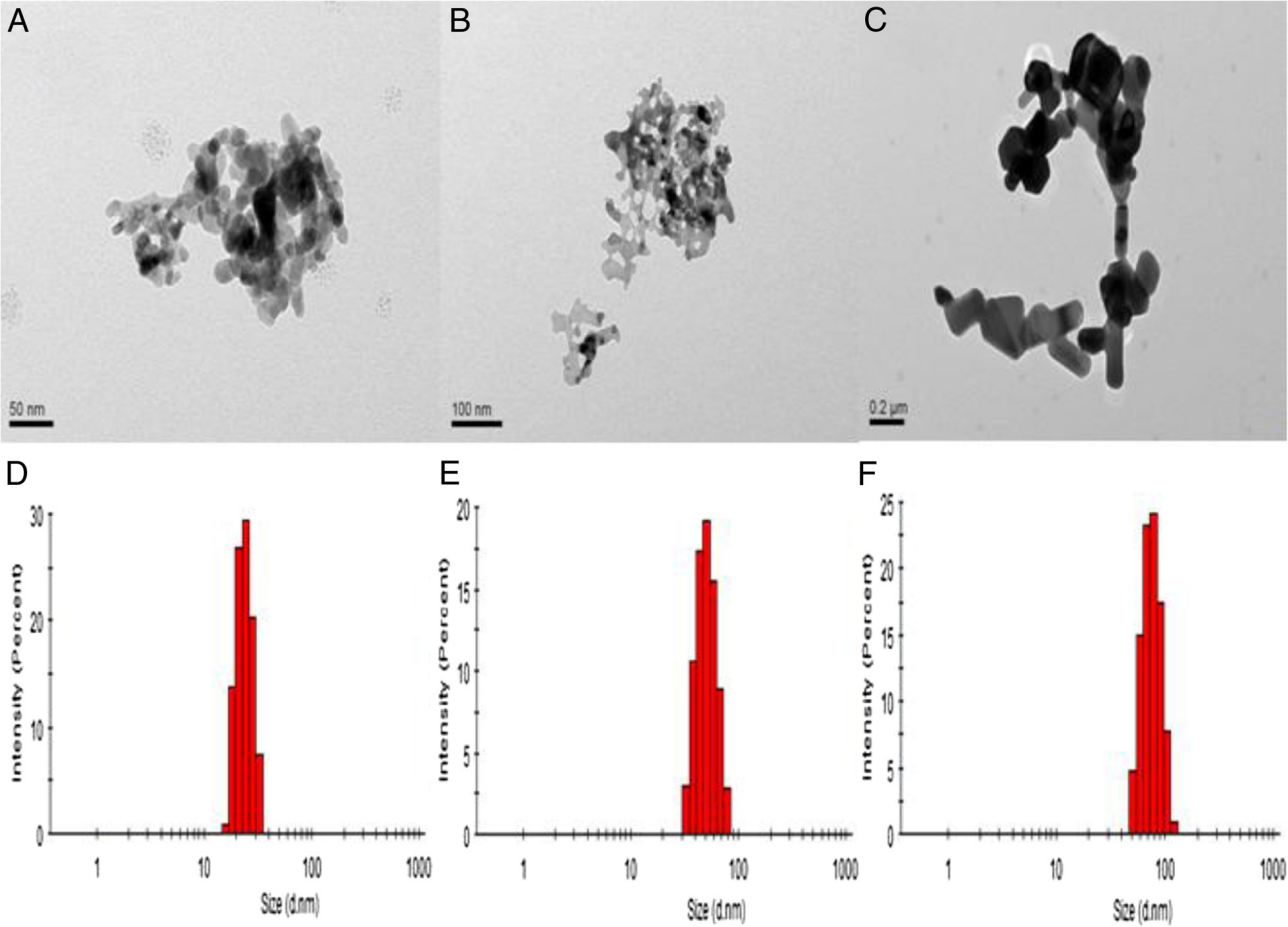
**Microscopy characterizations of ZnO NPs.** TEM images of an average **(A)** 26-nm ZnO NP, **(B)** 62-nm ZnO NP, and **(C)** 90-nm NP. Ranges **(D)** from 15 to 30 nm for a nanosphere, **(E)** from 30 to 70 nm for a nanorod, and **(F)** from 60 to 100 nm for a nanorod. TEM scale bars: **(A)** 50 nm, **(B)** 100 nm, and **(C)** 200 nm.

The evaluation of nanomaterials is based on their size, shape, and distribution. Size distribution was assessed using a Malvern instrument. Figure 1 shows representative transmission electron microscopy images of ZnO NPs. The results show the average particle diameter of ZnO NPs: 26.21 ± 11.14 nm (A), 62.42 ± 9.18 nm (B), and 90.81 ± 8.89 nm (C). Figure 1D shows the ranges from 15 to 30 nm for a nanosphere, Figure 1E from 30 to 70 nm for a nanorod, and Figure 1 F from 60 to 100 nm for a nanorod.

**Figure 2:**
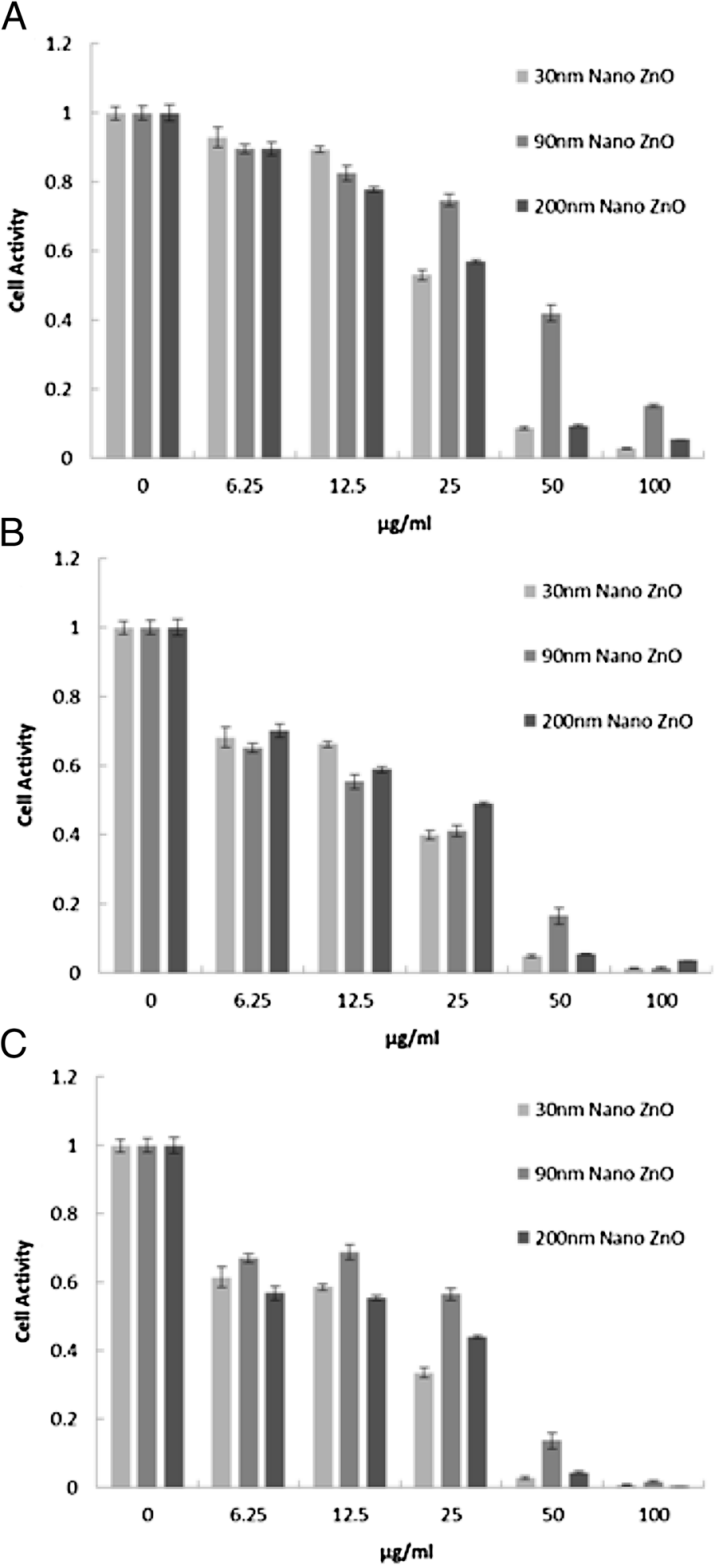
**Cytotoxicity of ZnO NPs on Caco-2 cells.** MTT assay. Cell viability of Caco-2 cells treated with different concentrations of different-sized ZnO NPs at different times. Exposure to ZnO NPs for **(A)** 12 h, **(B)** 24 h, and **(C)** 36 h. Results are expressed as the percentage of cell activity compared to the control. The data are presented as the mean ± SD of three independent experiments (*n* = 5).

To assess the cell activity, the intracellular dose of formazan was quantified. Three different sizes of NPs were tested over a 12-, 24-, and 36-h exposure. As shown in Figure 2, the MTT results demonstrated that higher concentrations and longer incubation times generated more serious cytotoxicity. It was observed that the cell activity is statistically significantly different between the concentrations of 12.5 and 50 μg/ml for 24 h. For the data regarding the exposure to 26-nm ZnO NPs for 12 h, the percentage (%) MTT reduction (relative to control) of Caco-2 cells observed at concentrations of 25 and 50 μg/ml was 41.02% and 91.3%, respectively. The percentage of reduction was 25.3% and 58.1% after exposure to 62-nm ZnO NPs, and reduction was 42.11% and 90.7% after exposure to 90-nm ZnO NPs (Figure 2A). The 24-h value was chosen to confirm the viability and accessibility of the cells and taken as the appropriate time for the following test system [18-20]. The relevant IC_50_ values on Caco-2 cells were 15.55 ± 1.19 μg/ml, 22.84 ± 1.36 μg/ml, and 18.57 ± 1.27 μg/ml.

**Figure 3:**
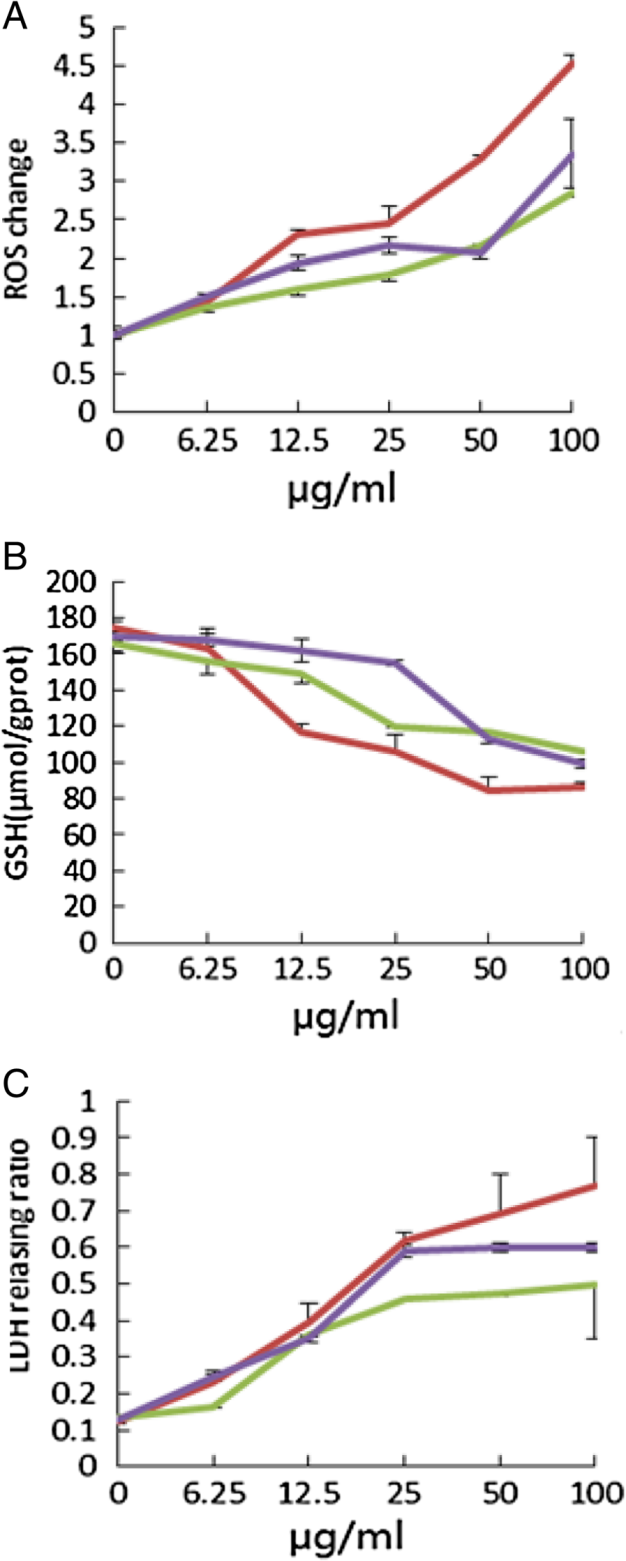
**The oxidative stress of ZnO NPs on Caco-2 cells.** Cell viability of Caco-2 cells treated with different concentrations of different-sized ZnO NPs for 24 h. The data are presented as the mean ± SD of three independent experiments (*n* = 5). **(A)** ROS change. **(B)** GSH detection. **(C)** LDH release. Red, 26-nm ZnO NPs; green, 62-nm ZnO NPs; violet, 90-nm ZnO NPs.

The results show an enhanced trend in ROS generation due to exposure to increased concentration of Caco-2 cells (Figure 3A). At low concentrations (around 6.25 μg/ml ZnO NPs), exposure to nano-ZnO resulted in a slight increase in intracellular ROS. The exposure at high concentrations (above 12.5 μg/ml ZnO NPs) results in significant increases in ROS. As for the exposure to 62-nm ZnO NPs for 24 h, the fold of ROS levels (relative to control) at concentrations of 6.25, 12.5, 25, 50, and 100 μg/ml was 1.35, 1.6, 1.8, 2.1, and 2.8, respectively. Intracellular ROS induced by 26-nm ZnO NPs at 100 μg/ml for 24 h reached 4.5-fold compared to the relative control cells. GSH is an antioxidant, preventing damage to important cellular components caused by reactive oxygen species such as free radicals and peroxides. As shown in Figure 3B, ZnO NPs significantly decreased the GSH level in Caco-2 cells compared with control values. Intracellular GSH was greatly reduced (117 ± 4 μmol/g prot) with 12.5 μg/ml of 26-nm ZnO NPs on Caco-2 cells, indicating functional damage from ROS; 26-nm and 62-nm ZnO NPs significantly decreased (106.1 ± 9 and 119.7 ± 0.4) intracellular GSH at 25 μg/ml, whereas at 100 μg/ml, a significant decrease occurred at both types tested. The colorimetric LDH release assay is a simple and robust method to assess cytotoxic effects on cells by measuring the activity of LDH in the cell culture supernatant. Figure 3C showed that ZnO induced a significant LDH release and thus loss of membrane integrity at both treatment concentrations. After a 24-h incubation, 25 μg/ml ZnO significantly increased LDH release in comparison to the controls. With 90-nm ZnO NPs, LDH release could be largely measured at 50 μg/ml. At less than 12.5 μg/ml, the 90-nm ZnO NPs did not show any membrane-damaging effects.

**Figure 4:**
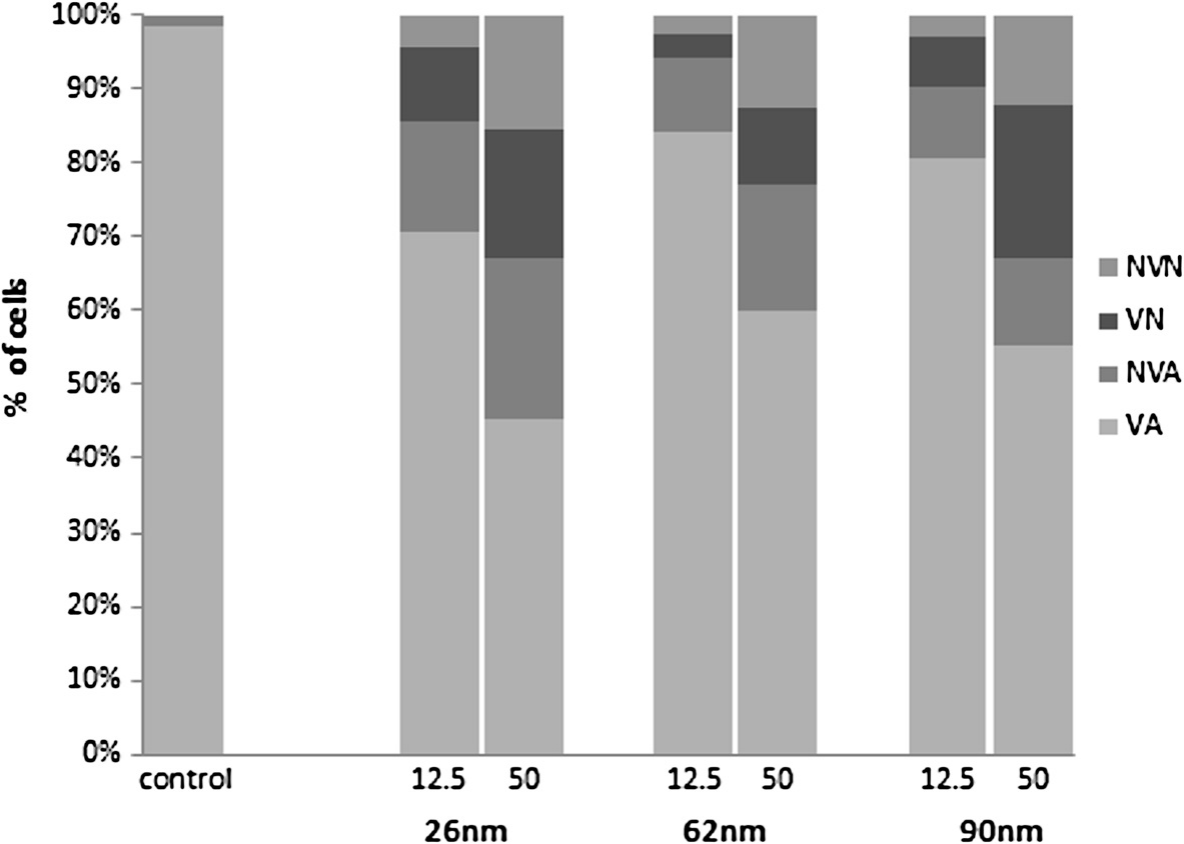
**Percentage of Caco-2 cells evaluated by AO/EB.** The data are presented as the mean of three independent experiments.

**Figure 5:**
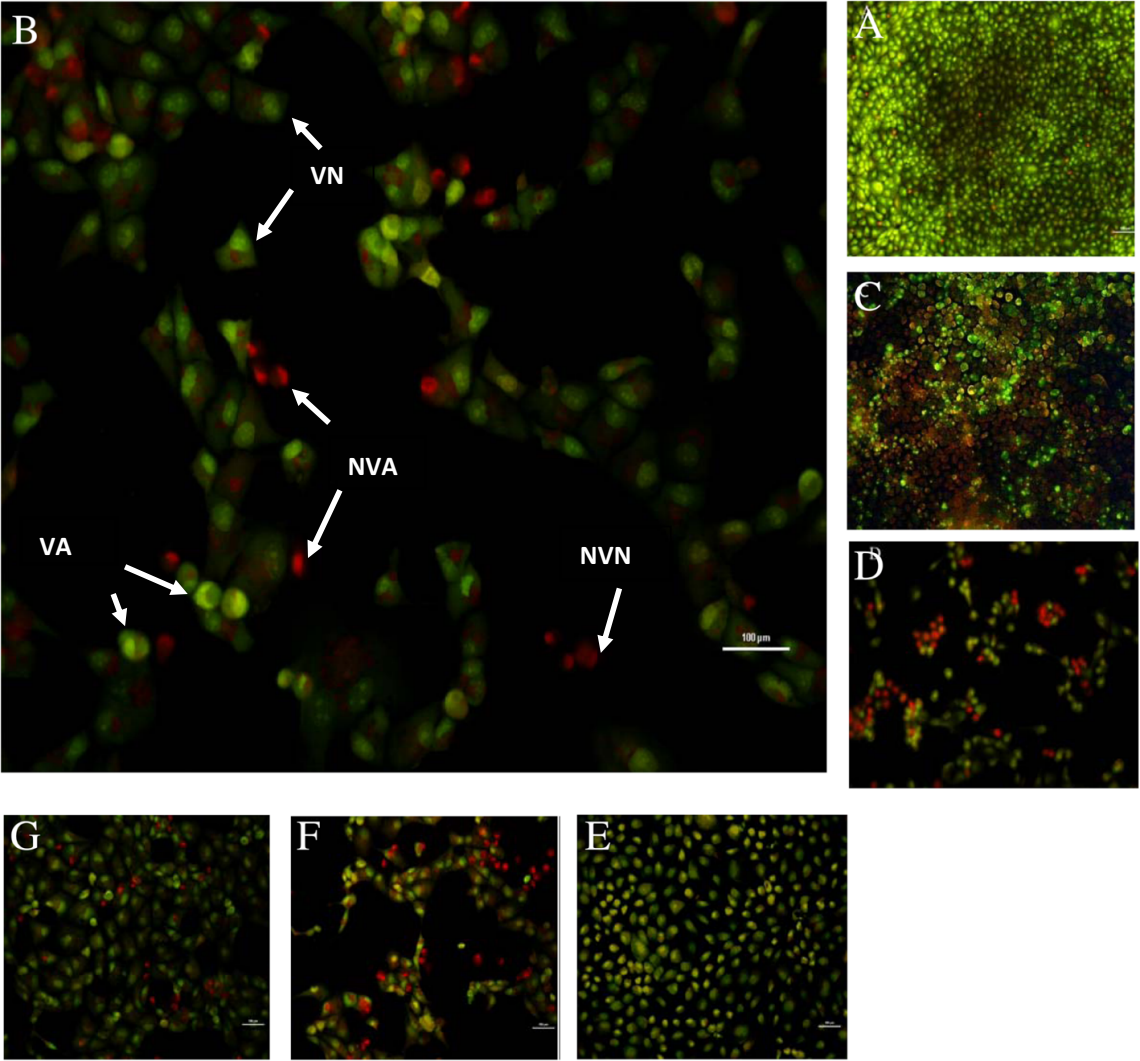
**Various morphologies of Caco-2 cells stained with AO/EB.** VN would have a uniform bright green nucleus and orange cytoplasm. VA, whose membranes are still intact but has started to cleave its DNA, would still have a green nucleus, but NVA, whose chromatin condensation becomes visible in the form of bright orange areas of condensed chromatin in the nucleus (EB predominates over AO), and NVN will have a uniform bright orange nucleus. **(A)** The control group, **(B)** 26-nm ZnO NPs at 50 μg/ml, **(C)** 26-nm ZnO NPs at 12.5 μg/ml, **(D)** 62-nm ZnO NPs at 50 μg/ml, **(E)** 62-nm ZnO NPs at 12.5 μg/ml, **(F)** 90-nm ZnO NPs at 50 μg/ml, and **(H)** 90-nm ZnO NPs at 12.5 μg/ml. VN, viable cell; VA, early apoptotic cell; NVA, late apoptotic cells; NVN, necrotic cell; EB, ethidium bromide; AO, acridine orange.

The acridine orange (AO)/ethidium bromide (EB) double staining principle combines the differential uptake of fluorescent DNA binding dyes acridine orange and ethidium bromide, and the morphological aspect of chromatin condensation in the stained nucleus [21]. The toxicity of ZnO NPs resulted in a dose-dependent decrease in the number of viable cells (VN) and a rise in early apoptotic cells (VA), late apoptotic cells (NVA), and necrotic cells (NVN) (Figure 4). The AO/EB assay is applicable for ZnO nanoparticles according to their cell membrane destabilization potential. Cultures exposed to 12.5 μg/ml ZnO NPs showed a decrease (70.5%, 84%, and 83% for 26-, 62-, and 90-nm ZnO NPs) in the number of viable cells when compared with the control (98.5%), with a concomitant increase in the number of early apoptotic cells (15%, 10%, and 10% for 26-, 62-, and 90-nm ZnO NPs). Cells exposed to a concentration of 50 μg/ml showed that late apoptotic cells and necrotic cells became the increasingly predominant cell type (Figure 5).

**Figure 6:**
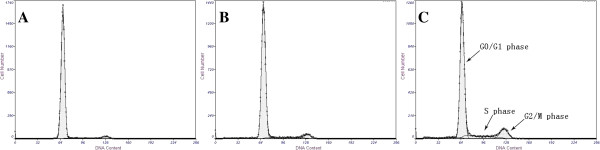
**PI fluorescence (DNA content) histograms of Caco-2 cells after exposure to ZnO NPs. (A)** Control culture (non-exposed). **(B)** Cells exposed to 26-nm ZnO NPs at 12.5 μg/ml. **(C)** Cells exposed to 26-nm ZnO NPs at 50 μg/ml. The data are presented as the mean ± SD of three independent experiments.

In Figure 6A, no abnormal DNA content was observed. The diploid was 94% in the G0/G1 phase, 3% in the S phase, and 2.93% in the G2/M phase. Figure 6B showed that the DNA content of cultures exposed to 26-nm ZnO NPs at 12.5 μg/ml was similar to the control group cells that were distributed to the G0/G1, S, and G2/M phases of the cell cycle. Figure 6C showed that the diploid was 78% in the G0/G1 phase, 11.1% in the S phase, and 10.8% in the G2/M phase. With an increase in the concentration, the percentage of cells during the G1 phase decreased significantly, the percentage of cells in the S phase was increasing, and the cells exposed to 50 μg/ml ZnO NPs during the G2 phase increased significantly. The same results happened with the cells exposed to 62-nm and 90-nm ZnO NPs. Our results clearly demonstrated that cells treated with ZnO NPs suffer the transition from G1 to S phase and from S to G2 phase. Once reaching the G2 phase, DNA damage is insufficient. There must be a replication of DNA on the damaged template to offset the toxic effect [22-24] (Table 1).

**Table 1:** PI staining (flow assay)

**ZnO NP scale (nm)**	**Concentration (μg/ml)**	**The cell cycle (%)**		
		**G0/G1 phase**	**S phase**	**G2 phase**
Control cell	0	94.07 ± 5.13	3 ± 1.03	2.93 ± 1.1
26 nm	12.5	88.43 ± 6.16	6.64 ± 2.3	4.93 ± 3.6
	50	77.95 ± 6.83	11.19 ± 3.09	10.87 ± 2.78
62 nm	12.5	91.07 ± 4.1	5.46 ± 1.33	3.47 ± 1.34
	50	82.6 ± 3.54	8.95 ± 5.03	8.45 ± 3.14
90 nm	12.5	90.32 ± 6.35	50.5 ± 1.08	4.63 ± 1.44
	50	79.26 ± 6.3	11.69 ± 4.24	9.05 ± 2.09

Results are shown as the mean ± SD (*n* = 3).

## Discussion

It is necessary to consider the possibility of cell type differences. We have observed that the same additional concentrations of nano-ZnO at the same size showed different results in L02 and HEK293 [10]. As we know, the Caco-2 monolayer is widely used across the pharmaceutical industry as an *in vitro* model of the human small intestine mucosa to predict the absorption of orally administered drugs. These cells would have to be grown so that the cells joined together to form tight junctions if they were growing in the intestine. Caco-2 cells are approximately 40 to 70 μm, spindle- or polygon-shaped (high cell density), with adherent cells growing as a confluent monolayer. With increasing doses of ZnO NPs (above 25 μg/ml), the cells started to shrink and lost adhesion to the cell culture plate. Multiple assays have been adopted to enable the homogeneous measurement that can serve as markers of cell viability, cytotoxicity, and apoptosis. IC_50_ values of three ZnO particles in Caco-2 cells were 15.55 ± 1.19 μg/ml, 22.84 ± 1.36 μg/ml, and 18.57 ± 1.27 μg/ml for 26-, 62-, and 90-nm ZnO NPs. ZnO NPs of 26 nm in diameter present the highest toxicity, and NPs of 62 nm also appear to be less toxic and lethal than the ZnO NPS of 90 nm in diameter. ZnO NPs of 26 nm, especially in high concentrations, could cause reduction of the G1 phase and an increase in the S phase and the G2 phase cells to repair damaged genes. The same concentrations of 62-nm and 90-nm ZnO NPs did not have significantly different toxicity.

A systematic study of the influence of size scale and distribution is critical to an understanding of the toxicity mechanism [25]. Two principal factors cause the properties of nanomaterials to differ significantly from other materials: increased relative surface area and quantum confinement effect. AshaRani et al. showed that the Ag nanoparticles in the range of 6 to 20 nm in diameter are small enough to pass though the plasma membrane and into the apical surface region of the cell, eventually gaining access to the nuclear DNA [26]. Huang et al. investigated the different free radical savaging efficiencies of nano-Se with different sizes: small size (5 ~ 15 nm), medium size (20 ~ 60 nm), and large size (80 ~ 200 nm). There was one potential size-dependent consequence of nano-Se on scavenging free radicals: small size and medium size had similar effects and were both better than the large size [27]. Dissimilar results were reported by Wang, who prove that there were no differences of GSH and LDH in cells supplemented with different sizes and concentrations of nano-Se particles. There is still little knowledge about the invisible details of ZnO toxicity related with the nanoparticle sizes, including how they are transported in cells and how nanoparticles interact with the cell membrane and organelles. In our study, ZnO nanoparticles that are dispersed in the culture medium and spread over the cell surface could only enter the cells via their apical surface. We conclude that even a low concentration of ZnO NPs has the potential to cause toxicity like other nanomaterials. Cell apoptosis and necrosis, oxidative stress, and cell cycle arrest raise the concern about the applications of ZnO NPs. On the other hand, not all nanomaterials have a particle size effect. It is suggested that 26-nm ZnO NPs appeared to have the highest toxicity, while a certain concentration of nano-ZnO with the average sizes of 62 nm and 90 nm had the same influence on the membrane integrity and cell cycle of Caco-2.

## Conclusions

The results revealed that cytotoxicity exhibited dose- and time-dependent effects for different kinds of ZnO NPs. ZnO induces oxidative stress, decreases viability, and increases cell death in Caco-2 cells. The 26-nm ZnO NPs appeared to have the highest toxicity. Different sizes of ZnO NPs could cause a significant reduction in GSH and with increase in ROS and LDH. ZnO could also cause reduction of the G1 phase and an increase in the S phase and the G2 phase cells to repair damaged genes, while no differences were obtained between 62-nm and 90-nm ZnO NPs. Finally, there is still little knowledge about the detail of ZnO toxicity related with the nanoparticle sizes, including how they are transported in cells and how nanoparticles interact with the cell membrane and organelles.

## Abbreviations

AO: Acridine orange; Caco-2 cells: Human epithelial colorectal adenocarcinoma cells; EB: Ethidium bromide; GSH: Glutathione; LDH: Lactate dehydrogenase; MTT: 3-(4,5-dimethylthiazol-2-yl)-2,5-diphenyltetrazolium bromide; NVA: Late apoptotic cells; NVN: Necrotic cell; PI: Propidium iodide; ROS: Reactive oxygen species; VA: Early apoptotic cell; VN: Viable cell; ZnO NPs: Zinc oxide nanoparticles.

## Competing interests

The authors declare that they have no competing interests.

## Authors’ contributions

RFG came up with the idea, contributed to the design of the experiment, and agreed with the paper’s publication. TSK and YJS conducted most of experiments that the manuscript mentioned and drafted the manuscript. XQC analyzed the data and drew the pictures. HJ and JZ revised the manuscript critically and made a few changes. All authors read and approved the final manuscript.
